# Cognitive Outcomes at 18 Months: Findings from the Early Life Interventions for Childhood Growth and Development in Tanzania (ELICIT) Trial

**DOI:** 10.4269/ajtmh.21-0596

**Published:** 2021-12-06

**Authors:** Tarina Parpia, Erling Svensen, Sarah Elwood, Anne Wanjuhi, Ladislaus Blacy, Eliwaza Bayo, Eric Houpt, Elizabeth Rogawski McQuade, Mark DeBoer, James Platts-Mills, Estomih Mduma, Rebecca Scharf

**Affiliations:** ^1^ University of Virginia Division of Infectious Diseases and International Health, Charlottesville, Virginia;; ^2^ Haukeland University Hospital, Bergen, Norway;; ^3^ Haydom Global Health Research Centre, Haydom Lutheran Hospital, Haydom, Tanzania;; ^4^ Department of Pediatrics, University of Virginia, Charlottesville, Virginia

## Abstract

Micronutrient deficiencies and enteric infections negatively impact child growth and development. We enrolled children shortly after birth in a randomized, placebo-controlled, 2 × 2 factorial interventional trial in Haydom, Tanzania, to assess nicotinamide and/or antimicrobials (azithromycin and nitazoxanide) effect on length at 18 months of age. Cognitive score at 18 months using the Malawi Developmental Assessment Tool (MDAT), which includes gross motor, fine motor, language, and social assessments, was a secondary outcome. Here, we present the MDAT results of 1,032 children. There was no effect of nicotinamide (change in development-for-age Z score [DAZ] −0.08; 95% CI: −0.16, 0) or antimicrobials (change in DAZ 0.04; 95% CI: −0.06, 0.13) on overall MDAT score. The interventions had no effect on cognitive outcomes in subgroups defined by gender, socioeconomic status, birthweight, and birth season or on MDAT subscores. Further analyses are needed to identify targetable risk factors for impaired cognitive development in these settings.

## INTRODUCTION

Early childhood malnutrition and enteric disease in limited-resource settings has both short- and long-term impacts, including poor cognitive development, lower educational attainment, and decreased economic productivity.^1^^,^^2^

Haydom, Tanzania, a rural, semiarid town in a resource-poor setting, is the site of the Early Life Interventions for Childhood Growth and Development in Tanzania (ELICIT) study, an interventional trial primarily assessing the effect of antimicrobials and nicotinamide on linear growth in children.^3^ Both diarrheal illness and enteropathogen burden significantly impact growth and cognitive development, with higher diarrheal burdens leading to deficits in verbal fluency and in some cases equating to a decrease of up to 10 IQ points.^4^ Children with high enteropathogen burden, independent of diarrhea, have poorer growth outcomes at 2 years.^5^

The region surrounding Haydom has a unimodal crop cycle and maize-predominant diet, making the inhabitants particularly susceptible to micro- and macronutrient deficiencies, including niacin/nicotinamide. Tryptophan is an essential amino acid, and niacin, part of the tryptophan–kynurenine–niacin pathway, is a precursor to nicotinamide adenine dinucleotide (NAD+). Lower serum levels of tryptophan were associated with short stature in children in Haydom, whereas lower kynurenine-to-tryptophan ratios were associated with poorer vaccine response.^6^ There are limited data examining the effect of nicotinamide on cognitive development. Mutations in this pathway have been associated with congenital malformations in human and mouse models. In mouse models, niacin supplementation prevented these defects^7^ and increased spatial learning ability.^8^ Finally, nicotinamide riboside supplementation given to postpartum mouse mothers improved physical and neurobehavioral development (enhanced spatial learning and motor learning).^9^

These interventions target two independent pathways for poor growth and development that we judged to be particularly relevant in this setting. Assessing them in a single factorial study also allowed for the evaluation of these interventions in synergy. Here, we estimate the effects of antimicrobial administration and nicotinamide supplementation on cognitive function in the ELICIT trial.

## MATERIALS AND METHODS

We have previously described the rationale, design, and baseline characteristics of this trial as well as effects on the primary outcomes.^3^^,^^10^^,^^11^ Briefly, mother/child dyads were enrolled within 14 days of the child’s birth from September 2017 to September 2018. Dyads were randomized to one of four arms: placebo plus placebo, azithromycin and nitazoxanide plus placebo, nicotinamide plus placebo, or azithromycin and nitazoxanide plus nicotinamide. Mothers received daily nicotinamide (or placebo) from birth through 6 months. Children received daily nicotinamide (or placebo) from 6 months onwards. Children received azithromycin (or placebo) at 6, 9, 12, and 15 months, and nitazoxanide (or placebo) at 12 and 15 months. In addition to the interventions, standardized questionnaires obtained at each monthly visit elicited information concerning breastfeeding practices, maternal and child illness, healthcare seeking behaviors, and maternal (once) and child (multiple) anthropometry. At the initial visit, household information was collected regarding family assets, ownership of animals, water, sanitation, and hygiene practices.^3^

The Malawi Development Assessment Tool (MDAT) used in this study was created to assess child development in children living in a rural sub-Saharan context.^12^^–^^16^ The MDAT is a relatively new test (published in 2010) with good reliability (94–100%).^12^ The cognitive team from The Etiology, Risk Factors, and Interactions of Enteric Infections and Malnutrition and the Consequences for Child Health (MAL-ED)^17^ and ELICIT studies worked together to adapt, pilot, and validate the MDAT locally for Haydom. The items, words, and tasks used in the assessment were similar to the original study environment in rural Malawi, where the assessment was developed. A group of children in the community not participating in the study piloted the assessment to determine feasibility, check each item, and train the field team. Six field workers completed training and practice over 4 weeks. Two were led by an official MDAT trainer from Kenya who spoke Swahili, and 2 additional weeks were by cognitive team members from the United States and Norway.

The assessment was translated to Swahili, then back translated into English by a separate interpreter to ensure translation accuracy. The assessment was delivered in Swahili or Iraqw, the first language for most participants. The fine and gross motor items did not require any adaptation and were used as developed in Malawi. Several questions in the language and social domains required adaptations for words not directly translatable to Swahili. Items were scored as 1 (pass) or 0 (not pass). Within each subtest, children completed items until they received a score of 0 for six items in a row.

All assessments were recorded on video, and each week the cognitive team watched 10–20% to provide feedback, avoid drift, and ensure consistency. Each assessment had three cognitive assessment field workers present: one to interact with the child, one to hand supplies and keep track of toys, and one to video; therefore, field team members could support one another inaccurate test administration.

All analyses were prespecified according to statistical analysis plan (found at https://clinicaltrials.gov/ct2/show/NCT03268902). The modified intention to treat (mITT) group, our primary analysis population, included all children with a valid MDAT assessment at the end of the study (18 months). The per-protocol group included children with any breastfeeding through age 6 months, those who received all doses of azithromycin and the initial nitazoxanide dose with no more than one of these outside the 14-day window around the target date, and those who received at least 50% of nicotinamide doses by pill and sachet counting.

We prespecified fine motor and language subsets as the primary cognitive outcomes because we hypothesized these subsets would be more closely associated with overall cognitive skills and academic readiness than gross motor and social subsets. Standardized MDAT Z-scores were calculated using version 1.1 of the MDAT Scoring Application in Shiny ( https://kieran-bromley.shinyapps.io/mdat_scoring_shiny/). Internal validity checks with correlated answers were performed for each section. We estimated associations between interventions and cognitive outcomes using linear regression, adjusted for prespecified covariates with *P* < 0.2 or a difference of ≥ 0.2 Z-score in a bivariable analysis. We compared nicotinamide versus no nicotinamide, antimicrobials versus no antimicrobials, and assessed the interactions between these interventions with a likelihood ratio test. The adjusted analyses were repeated for prespecified subgroups: birthweight, gender, birth season, socioeconomic status, maternal education, and birth order. Statistical analysis was performed using R version 1.2.1335 (R Foundation for Statistical Computing, Vienna, Austria, https://www.R-project.org/).

From May 3, 2019 to July 8, 2019, after enrollment was completed, this study was suspended by the Tanzanian regulatory board as a result of concerns over timing of laboratory testing and number of serious adverse events.^3^ These concerns were resolved and the study was resumed; however, 15% of 18-month MDATs were completed late. Therefore, all analyses were adjusted for age.

## RESULTS

**Figure 1.**
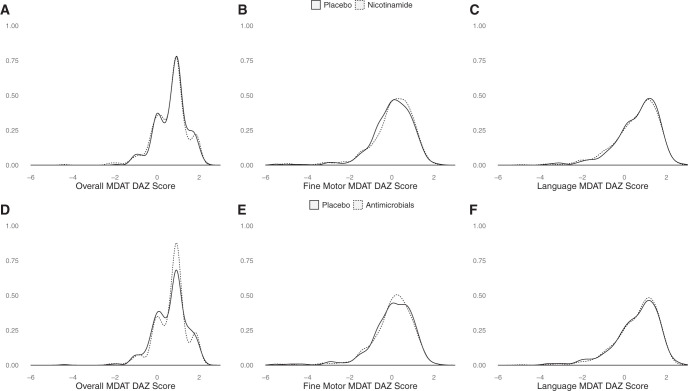
Distribution of Malawi Developmental Assessment Tool (MDAT) development-for-age Z-scores (DAZ) at 18 months by (**A**, **B**, and **C**) nicotinamide and (**D**, **E**, and **F**) antimicrobial intervention groups.

**Figure 2.**
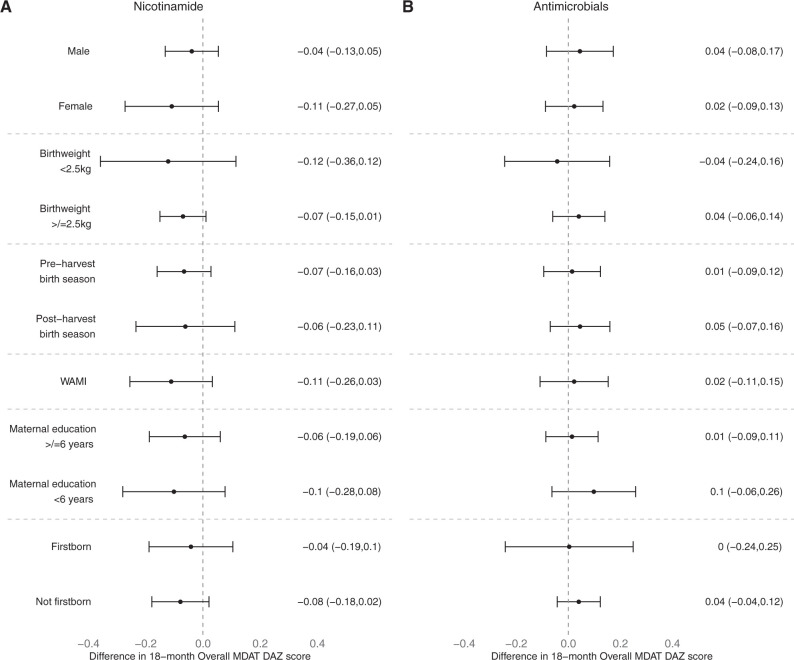
Effects of (**A**) nicotinamide and (**B**) antimicrobial interventions on Malawi Developmental Assessment Tool (MDAT) development-for-age Z-scores in subgroups defined by baseline sociodemographic characteristics.

In total, 1,188 children were enrolled and randomized, of whom 1,032 (86%) underwent MDAT testing at 18 months of age and were included. The score distribution was similar between active and placebo arms for both interventions for the overall MDAT as well as the fine motor and language subscores (Figure 1). The mean (±SD) overall, fine motor, and language MDAT development-for-age Z (DAZ) scores were 0.67 (±0.77), −0.01(±1.14), and 0.61 (±1.02), respectively. There was no association between MDAT DAZ score and nicotinamide or antimicrobial supplementation (Table 1). There was no statistical interaction between the nicotinamide and the antimicrobial interventions (*p*_(heterogeneity)_ = 0.2). In multivariable analyses, there was no significant association between overall MDAT DAZ score and study interventions by subgroup (Figure 2). Subgroup analyses for fine motor and language DAZ scores similarly showed no associations. In the per-protocol group (*N* = 959), we also found no association between MDAT DAZ score and nicotinamide or antimicrobial supplementation (data not shown).

**Table 1:** Effect of primary interventions on overall, fine motor, and language MDAT DAZ scores in the ELICIT trial

Intervention	Overall MDAT DAZ score		Fine motor MDAT DAZ score		Language MDAT DAZ score	
	Unadjusted	Adjusted	Unadjusted	Adjusted	Unadjusted	Adjusted
	Estimate (95% CI)		Estimate (95% CI)		Estimate (95% CI)	
Nicotinamide*	−0.07 (−0.16, 0.02)	−0.08 (−0.16, 0)	−0.01 (−0.15, 0.13)	0 (−0.06, 0.05)	−0.06 (−0.19, 0.06)	−0.08 (−0.19, 0.02)
Antimicrobials*	0.04 (−0.05, 0.13)	0.04 (−0.06, 0.13)	0.04 (−0.10, 0.17)	0.02 (−0.10, 0.15)	0.03 (−0.09, 0.16)	0.03 (−0.08, 0.13)

DAZ = development-for-age Z; ELICIT = Early Life Interventions for Childhood Growth and Development in Tanzania; MDAT = Malawi Developmental Assessment Tool. Overall scores were adjusted for: Sex, Water and sanitation, Assets, Maternal education, household Income (WAMI), maternal age, Datoga, mother school years, birth month, born in hospital, ward, MDAT age (days), enrollment weight-for-age Z score (WAZ), enrollment head circumference-for-age Z-score (HCZ), and enrollment length-for-age Z-score (LAZ). Fine motor scores were adjusted for: WAMI, mother height, Datoga, maternal age, maternal education > 7, birth month, MDAT age (days), firstborn, and enrollment WAZ. Language scores were adjusted for: Sex, WAMI, Datoga, mother school years, birth month, born in hospital, MDAT age (days), ward, enrollment WAZ, and enrollment HCZ.

* Values are in reference to placebo.

## DISCUSSION

In this seasonal subsistence community with a heavy maize-based diet and a high burden of enteric pathogens,^5^ we did not find any effect of daily nicotinamide or scheduled antimicrobials on cognitive outcomes. There are several possible reasons why daily nicotinamide failed to improve cognitive outcomes. First, dosing or bioavailability from breast milk may be insufficient to overcome the malnutrition faced early in life. Second, nicotinamide deficiency may not be present despite the area’s maize predominant diet. Alternatively, supplementation of deficiencies in this pathway may not significantly affect learning and cognitive development despite mouse models demonstrating so.^7^^–^^9^ Similarly, the dosing frequency or timing of antimicrobial supplementation received may not be sufficient to overcome predisposing environmental factors, prevent pathogen-associated changes in gut function, or successfully eradicate carriage of these pathogens. The treatment of enteric infections is the rationale for antimicrobial interventions, but infections are hard to prevent in areas of high transmission. The Water quality, Sanitation conditions, and Caregiver handwashing practices (WASH) Benefits study in Bangladesh^18^ and the Sanitation Hygiene Infant Nutrition Efficacy (SHINE) study in Zimbabwe^15^ showed that improved water treatments, sanitation, and handwashing interventions did not affect pathogen carriage or child growth. In Zimbabwe, there was no significant improvement in MDAT scores in the WASH intervention group,^15^ whereas in Bangladesh, effects on cognitive development have not yet been analyzed.

This study benefited from a large study population with little drop out in an area known to have high rates of malnutrition and childhood stunting. Additionally, the MDAT has good cultural validity^12^ and has been used broadly as an assessment of early cognitive development, largely in East Africa. The findings from our per-protocol group did not differ from the mITT group. Limitations include giving a single micronutrient, and antimicrobials at limited intervals, which may not sufficiently treat infections or sustainably change micronutrient status. Additionally, some children may have received antimicrobials from local pharmacies, resulting in effective crossover between antimicrobial arms and no appreciable effect of the intervention.

Although there was a delay in MDAT assessments for some children, the delay did not differ by intervention arm (Supplemental Table 1) and we adjusted for age at assessment. Cognitive testing at 18 months is early in a child’s life and later assessments may be more informative.

Although we did not find an effect of scheduled antimicrobial or nicotinamide supplementation on cognitive outcomes at 18 months, this large, well-characterized cohort of children can be used to identify pre- and postnatal targets for interventions to improve cognitive development. Planned analyses of routinely collected breast milk, stool, and blood samples will assess the relationship between enteropathogens, breast milk composition, and enteric dysfunction on cognitive development. Future analysis will also include observational assessment of additional factors affecting cognitive development such as maternal characteristics, low birthweight, pathogen burden, and birth season.

## Supplemental Material


Supplemental materials
