# Supplementary material for: Cognitive Outcomes at 18 Months: Findings from the Early Life Interventions for Childhood Growth and Development in Tanzania (ELICIT) Trial
Source: Am J Trop Med Hyg. 2021 Dec 6;106(2):441–5. doi: 10.4269/ajtmh.21-0596 (PMC8832914; doi:10.4269/ajtmh.21-0596)
Supplement: Supplementary file 1 [file tpmd210596.SD1.pdf]

Supplemental Table 1: Baseline characteristics of the modified intention to treat (mITT) group by intervention arm.*

	**Arm 1 (n = 264)**	**Arm 2 (n = 267)**	**Arm 3 (n = 250)**	**Arm 4 (n = 251)**
**Intervention assignment**				
Antimicrobial arm	Placebo	Active	Placebo	Active
Nicotinamide arm	Placebo	Placebo	Active	Active
**Sociodemographics**				
Child age (days)	5.9 ± 3.4	5.9 ± 3.7	5.8 ± 3.7	5.9 ± 3.6
Female sex	122 (46.2)	129 (48.3)	122 (48.8)	123 (49)
Hospital birth	145 (54.9)	142 (53.2)	121 (48.4)	131 (52.2)
Birth order (median; min, max)	3 (1, 12)	4 (1, 12)	4 (1, 12)	3 (1, 12)
Iraqw tribe	212 (80.3)	225 (84.3)	203 (81.2)	206 (82.1)
Maternal age	28.07 ± 7.12	27.92 ± 6.79	27.89 ± 6.19	27.71± 6.58
Maternal height (cm)	157.82 ± 5.5	157.09 ± 5.5	157.34 ± 5.74	157.16 ± 5.94
Mother with ≥ 7 years of education	215 (81.4)	190 (71.2)	192 (76.8)	188 (74.9)
Monthly income (/1000TSH)	51.3 ± 67.7	46.4 ± 41.8	48.8 ± 49.5	49.3 ± 44.3
**Risk Factors**				
Access to an improved drinking water source	174 (65.9)	175 (65.5)	169 (67.6)	165 (65.7)
Drinking water > 10 minutes from home	217 (82.2)	215 (80.5)	206 (82.4)	200 (79.7)
Access to an improved latrine	35 (13.3)	22 (8.2)	26 (10.4)	32 (12.7)
Agricultural land ownership	255/264 (96.6)	260/267 (97.4)	243/250 (97.2)	245/251 (97.6)
Water, Assets, Maternal Education, and Household Income Index score (Median ± IQR)	0.3 ± 0.1	0.3 ± 0.1	0.3 ± 0.1	0.3 ± 0.1
**Anthropometry**				
Enrollment length in cm (SD)	49.27 ± 2.02	48.78 ± 2.13	49 ± 2.11	49.1 ± 2.07
range	min: 42.2, max: 59	min: 40, max: 57	min: 41, max: 55	min: 38, max: 57
Enrollment weight in kg (SD)	3.15 ± 0.47	3.12 ± 0.46	3.12 ± 0.48	3.15 ± 0.51
range	min:1.82, max: 4.6	min: 1.9, max: 4.7	min: 1.58, max: 4.4	min: 1.6, max: 5.72
Enrollment head circumference in cm (SD)	34.74 ± 1.3	34.59 ± 1.33	34.81 ± 1.35	34.78 ± 1.3
range	min: 31.3, max: 38.2	min: 30, max: 38.4	min: 28.3, max: 38.5	min: 31.5, max: 40

* Mean ± standard deviation is shown for continuous variables and number (percentage) for dichotomous variables unless otherwise stated
